# Hepatocyte growth factor pretreatment boosts functional recovery after spinal cord injury through human iPSC-derived neural stem/progenitor cell transplantation

**DOI:** 10.1186/s41232-023-00298-y

**Published:** 2023-10-16

**Authors:** Yu Suematsu, Narihito Nagoshi, Munehisa Shinozaki, Yoshitaka Kase, Yusuke Saijo, Shogo Hashimoto, Takahiro Shibata, Keita Kajikawa, Yasuhiro Kamata, Masahiro Ozaki, Kaori Yasutake, Tomoko Shindo, Shinsuke Shibata, Morio Matsumoto, Masaya Nakamura, Hideyuki Okano

**Affiliations:** 1 Department of Orthopaedic Surgery, Keio University School of Medicine, 35 Shinanomachi, Shinjuku-Ku, Tokyo, 160-8582 Japan; 2 Department of Physiology, Keio University School of Medicine, 35 Shinanomachi, Shinjuku-Ku, Tokyo, 160-8582 Japan; 3 Department of Clinical Regenerative Medicine, School of Medicine, Fujita Health University, 1-98 Dengakugakubo, Kutukake-Cho, Toyoake-Shi, Aichi, 470-1192 Japan; 4 Electron Microscope Laboratory, Keio University School of Medicine, 35 Shinanomachi, Shinjuku-Ku, Tokyo, 160-8582 Japan; 5 Division of Microscopic Anatomy, Graduate School of Medical and Dental Sciences, Niigata University, 1-757 Asahimachi-Dori, Chuo-Ku, Niigata, Niigata 951-8510 Japan

**Keywords:** Cell transplantation therapy, Combination therapy, Hepatocyte growth factor, iPS cells, Neural stem cells, Neuroregeneration, Spinal cord injury

## Abstract

**Background:**

Human induced pluripotent stem cell-derived neural stem/progenitor cell (hiPSC-NS/PC)-based cell transplantation has emerged as a groundbreaking method for replacing damaged neural cells and stimulating functional recovery, but its efficacy is strongly influenced by the state of the injured spinal microenvironment. This study evaluates the impact of a dual therapeutic intervention utilizing hepatocyte growth factor (HGF) and hiPSC-NS/PC transplantation on motor function restoration following spinal cord injury (SCI).

**Methods:**

Severe contusive SCI was induced in immunocompromised rats, followed by continuous administration of recombinant human HGF protein into the subarachnoid space immediately after SCI for two weeks. Acute-phase histological and RNA sequencing analyses were conducted. Nine days after the injury, hiPSC-NS/PCs were transplanted into the lesion epicenter of the injured spinal cord, and the functional and histological outcomes were determined.

**Results:**

The acute-phase HGF-treated group exhibited vascularization, diverse anti-inflammatory effects, and activation of endogenous neural stem cells after SCI, which collectively contributed to tissue preservation. Following cell transplantation into a favorable environment, the transplanted NS/PCs survived well, facilitating remyelination and neuronal regeneration in host tissues. These comprehensive effects led to substantial enhancements in motor function in the dual-therapy group compared to the single-treatment groups.

**Conclusions:**

We demonstrate that the combined therapeutic approach of HGF preconditioning and hiPSC-NS/PC transplantation enhances locomotor functional recovery post-SCI, highlighting a highly promising therapeutic strategy for acute to subacute SCI.

**Supplementary Information:**

The online version contains supplementary material available at 10.1186/s41232-023-00298-y.

## Background

Spinal cord injury (SCI) can cause severe motor and sensory damage, substantially reducing quality of life. Our previous research has shown good motor functional recovery in animal models of SCI after the transplantation of human induced pluripotent stem cell-derived neural stem/progenitor cells (hiPSC-NS/PCs) [1–3]. Furthermore, we recently conducted the first human clinical trial of hiPSC-NS/PC transplantation for subacute SCI [4]. However, animal studies have indicated that the effectiveness of NS/PC transplantation therapy varies depending on the severity of the injury, with reduced therapeutic effects observed in severe models [1–6]. These limitations in the regenerative capacity of NS/PC transplantation therapy pose major obstacles to achieving even more successful clinical applications.

One of the key factors contributing to these limitations is the poor preservation of spinal cord tissue after injury [6, 7]. Moreover, the survival of transplanted cells can be compromised by avascular regions and the inflammatory response induced by secondary damage, even at the subacute stage [6, 8]. These factors hinder neural regeneration at the injury site and impede the potential reorganization of neural connections between host and graft neurons. Therefore, the development of a strategy that improves the environment of the injured spinal cord to enhance the efficacy of NS/PC transplantation is crucial.

Hepatocyte growth factor (HGF) is a highly promising clinically relevant candidate for enhancing the efficacy of NS/PC transplantation. HGF is a potent growth factor that promotes tissue regeneration through the MET receptor [9–13]. HGF-MET signaling activation not only has anti-inflammatory effects but also promotes cell proliferation, neuronal differentiation, and proangiogenic actions and exhibits antiapoptotic and immunomodulatory properties [11, 14–17]. Previous studies have demonstrated that HGF administration enhances motor function following acute SCI in various animal models of SCI [18–20]. Furthermore, a clinical trial involving the administration of recombinant human HGF protein to patients with acute human SCI showed improvement in motor function and safety [21]. HGF achieves these positive effects by preserving nerve tissue in the injured area through neuroprotective actions such as anti-inflammatory effects and angiogenesis. As such, we propose that HGF treatment, with its ability to improve the spinal cord environment after SCI, could offer a distinct advantage in enhancing the efficacy of hiPSC-NS/PC transplantation.

The objective of the present study was to investigate the impact of a combined therapeutic approach involving HGF and hiPSC-NS/PC transplantation on the restoration of motor function after SCI. In addition to investigating the mechanisms by which the positive effects of HGF are obtained, we also aimed to assess any potential risks associated with hiPSC-NS/PCs including tumorigenicity [22]. This study demonstrates that HGF pretreatment is a powerful therapeutic strategy to promote nerve regeneration in hiPSC-NS/PC transplants and provides valuable scientific insights into the mechanism involved.

## Results

### HGF treatment of SCI suppresses inflammatory responses and stimulates the regenerative process in acute SCI

**Fig. 1:**
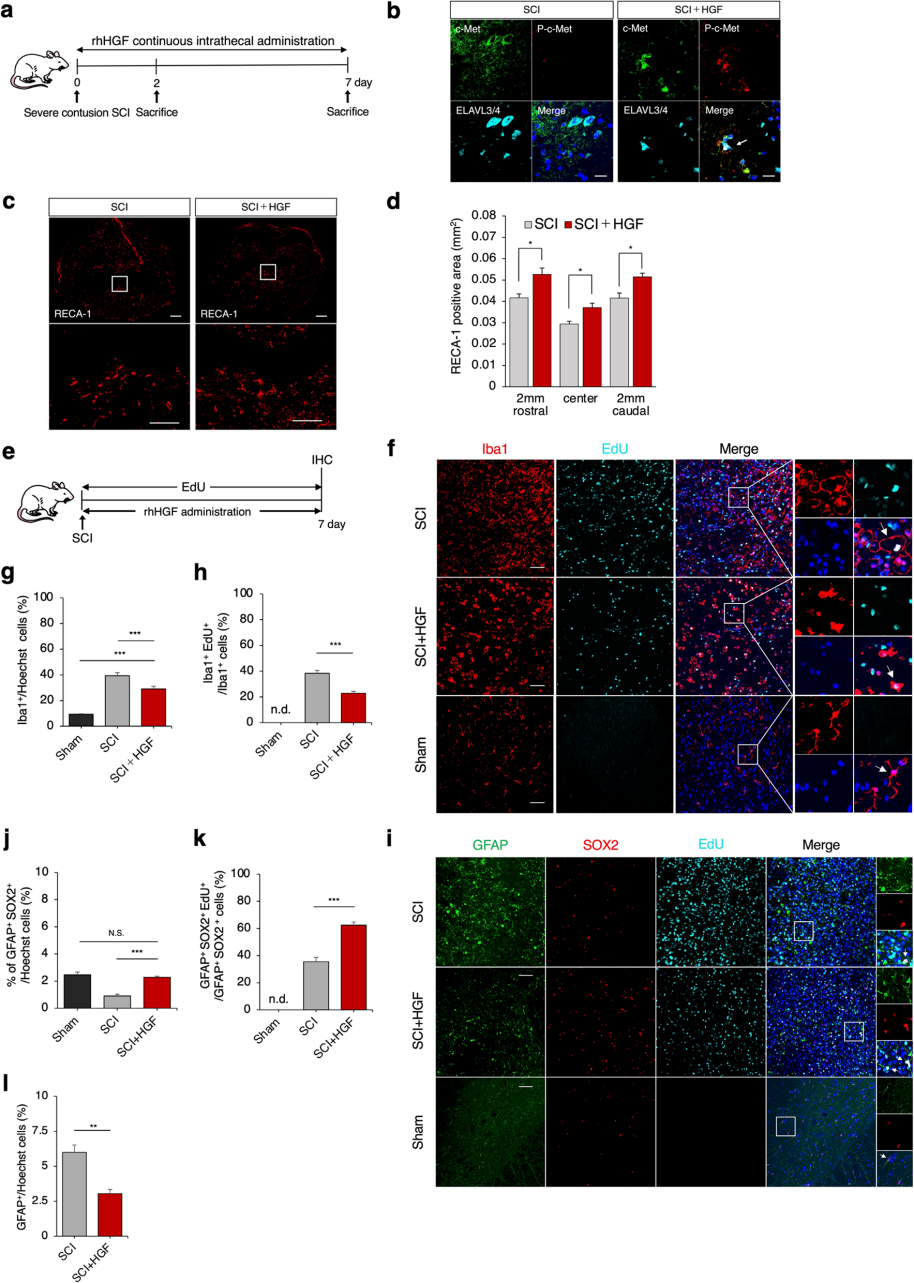
HGF-induced promotion of vascularization, suppression of microglial activation, and proliferation of endogenous NSCs in acute SCI. **a** Experimental design for analyzing microenvironmental changes in acute SCI. **b** Analysis of cell activation by rhHGF around the lesion site at 2 days after SCI by immunocytochemical staining of c-Met, pc-Met, and ELAVL3/4 in axial sections. Scale bars, 20 μm. **c** Representative images of axial sections stained for RECA-1 at the lesion epicenter and 2 mm rostral and caudal sites in each group at 7 days after SCI. Scale bars: 50 and 20 μm (enlarged images). **d** Quantification of the RECA-1^+^area in axial sections at each level. *n* = 5 each. *p < .05 according to the Mann‒Whitney U test. **e** Experimental design for analyzing the proliferation of Iba1^+^ microglia, GFAP^+^ astrocytes, and GFAP^+^SOX2^+^ NSCs. **f** Representative images of axial sections stained for Iba1 and EdU at the lesion epicenter in each group at 7 days after SCI. Arrowheads show examples of various forms of Iba1^+^ microglia. Scale bars: 50 μm. **g** Quantification of Iba1^+^ cells in axial sections. *n* = 5 each. *p < 0.05, **p < 0.01, ***p < 0.001 according to one-way ANOVA followed by the Tukey–Kramer test. **h** Quantification of Iba1^+^EdU^+^ cells in axial sections. *n* = 5 each. *p < 0.05, **p < 0.01, ***p < 0.001 according to the Mann‒Whitney U test. **i** Representative images of axial sections stained for GFAP^+^, SOX2^+^, and EdU^+^ cells at the lesion epicenter in each group at 7 days after SCI. Scale bar: 50 μm. **j**, **k** Quantification of GFAP^+^SOX2^+^ and GFAP^+^SOX2^+^EdU^+^ cells in axial sections. *n* = 5 each. *p < 0.05, **p < 0.01, ***p < 0.001 according to one-way ANOVA followed by the Tukey–Kramer test. **l** Quantification of GFAP^+^ cells in axial sections. *n *= 5 each. *p < 0.05, **p < 0.01 according to the Mann‒Whitney U test. In all graphs, the data are presented as the mean ± SEM. n.d.; not detected

To investigate the therapeutic effects of HGF on the acute SCI model, we directly administered recombinant human HGF protein (rhHGF) to the center of the injury immediately after SCI via a catheter connected to an osmotic mini-pump for the severe acute contusion SCI model (see Methods section for details) (Fig. 1a, Supplementary Fig. 1a-c). First, we performed immunohistochemical staining of c-Met (c-Met) and phosphorylated-c-Met (p–c-Met) receptors in the SCI (PBS) and SCI + HGF (HGF) groups to verify that the administered HGF was acting on neurons. The results revealed more p–c-Met-expressing cells in the SCI + HGF group than in the SCI group (Fig. 1b). Vascularization was assessed through rat endothelial cell antigen-1 (RECA-1) immunostaining. Quantitative analysis of axial cross-sections showed a significantly increased RECA1-positive area in the epicenter, rostral and caudal regions of the lesions in the SCI + HGF group compared to the SCI group (Fig. 1c, d; epicenter: 0.037 mm^2^ ± 0.002 mm^2^ vs. 0.029 mm^2^ ± 0.001 mm^2^, *p* = 0.032; rostral: 0.052 mm^2^ ± 0.003 mm^2^ vs. 0.041mm^2^ ± 0.001 mm^2^, *p* = 0.016; caudal: 0.052 mm2 ± 0.001 vs. ± 0.002, *p* = 0.032). These results suggest that HGF administration enhanced vascularization around the injured site in acute SCI.

To examine activated microglia that contribute to inflammation around the lesion, we used 5-ethynyl-2′-deoxyuridine (EdU) labeling and immunohistochemical staining for the microglial marker ionized calcium binding adaptor molecule 1 (Iba1) (Fig. 1e, f). In the sham (non-SCI) group, we observed approximately 9% Iba1 + cells (Iba1 + /Hoechst %) and no Iba1^+^EdU^+^ cells. In contrast, the SCI + HGF group showed a significant decrease in the number of Iba1^+^ and Iba1^+^EdU^+^ cells (29.1% ± 1.9% vs. 39.4% ± 2.3%, *p* < 0.001; 22.9% ± 1.4% vs. 38.4% ± 2.2%, *p* < 0.001) compared to the SCI group (Fig. 1g, h). In addition, the morphology of the Iba1-positive microglia differed between the groups: in the SCI group, microglial proliferation was strongly increased, and the microglia had a large bulging shape (Fig. 1f), in contrast to the normal microglial morphology. In the sham group, the microglia were ramified in shape, while in the HGF + SCI group, they were amoeboid in shape. The infiltration of macrophages should also be considered the site of injury, but in any case, the abnormalities in the proliferation and morphology of Iba1-positive cells were ameliorated by HGF administration (Fig. 1f-h). Collectively, the SCI + HGF group exhibited a significant decrease in the number of Iba1^+^ and Iba1^+^EdU^+^ cells compared to the SCI group. The morphology of Iba1^+^ microglia differed between groups. These abnormalities in Iba1^+^ cell proliferation and morphology were mitigated by HGF administration.

We further studied the changes in endogenous neural stem cells (NSCs) and reactive astrocytes around the lesion site using the astrocyte marker glial fibrillary acidic protein (GFAP) and the NSC marker sex-determining region Y-box 2 (SOX2) (Fig. 1i-l). It was reported that endogenous NSCs, identified by co-expression of GFAP and SOX2, contribute to nerve regeneration after central nervous systems (CNS) injury [23, 24]. The SCI + HGF group had significantly more GFAP^+^SOX2^+^ and GFAP^+^SOX2^+^EdU^+^ cells than the SCI group (2.2% ± 0.07% vs. 0.9% ± 0.13%, *p* < 0.001; 62.4% ± 2.3% vs. 35.4% ± 3.1%, *p* < 0.001) (Fig. 1j, k). Moreover, significantly fewer GFAP^+^ cells were found in the SCI + HGF group than in the SCI group (5.9% ± 0.52% vs. 3.0% ± 0.29%, *p* = 0.008) (Fig. 1l). These results suggest that administering HGF after SCI may enhance the proliferation of endogenous NSCs and suppress reactive astrocytes.

**Fig. 2:**
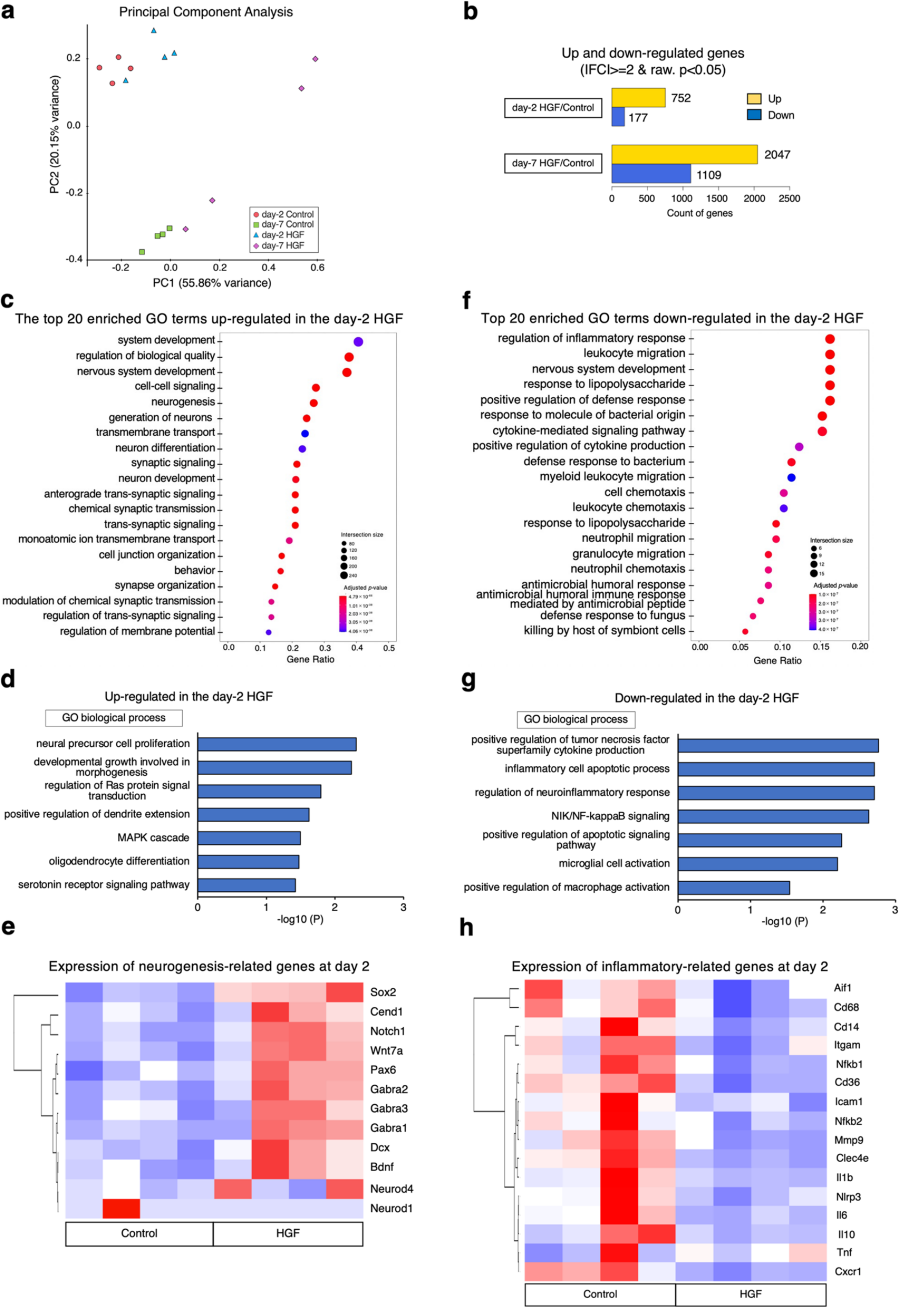
Transcriptome analysis of HGF-induced microenvironmental changes in the spinal cord. **a** Principal component analysis of the gene expression data in rats at 2 days and 7 days after SCI, with or without HGF administration. The spatial arrangement of the points in the plot reflects the overall data similarity between the samples. x-axis, principal component (PC) 1 (55.86%); y-axis, PC2 (20.15%). **b** Differentially expressed gene (DEG) profile investigation using RNA-seq. Significant genes were selected with a cutoff, *p* < 0.05, and fold change > 2.0. day-2 HGF vs. day-2 control comparison: 929 DEGs, 752 upregulated, 177 downregulated; day-7 HGF vs. day-7 control: 3156 DEGs, 2047 upregulated, 1109 downregulated. **c**, **d** Dot plot showing the top 20 most significant GO biological process terms associated with upregulated expression in the day-2 HGF group compared with the day-2 control group (upper row). The enriched GO terms of the biological process are increased in the HGF group (lower row). **e** Heatmap of the expression values of neurogenesis-related genes in the day-2 control and HGF groups. Scale: z score of gene expression. **f**, **g** Dot plot showing the top 20 most significant GO biological process terms associated with downregulated expression in the day-2 HGF group compared with the day-2 control group (upper row). The enriched GO terms of the biological process are decreased in the HGF group (lower row). **h** Heatmap of the expression values of inflammatory-related genes in the day-2 control and HGF groups. Scale: z score of gene expression. Adjusted *p* value: adjusted *p* value by FDR, Intersection size: The number of unique DEGs that are annotated to the term ID. Gene ratio: calculated as the ratio of intersection size and query size

Next, we performed transcriptome analysis to assess the molecular impact of HGF administration on the spinal cord microenvironment during the acute phase of SCI (day 2 HGF; day 7 HGF; day 2 Control; day 7 Control). Principal component analysis revealed that rat gene expression profiles in the spinal cord were clustered regarding both the time after SCI and with or without HGF administration (Fig. 2a). When comparing the HGF and control groups on days 2 and 7, we identified 929 (day 2) and 3156 (day 7) genes with significant differences in expression [*p* < 0.05, log_2_fold change (FC) > 1] (Fig. 2b). Gene Ontology (GO) analysis of the regulated genes indicated enhancement of many neurogenesis-related terms (Fig. 2c, Supplemental Fig. 2a). Other GO terms such as "neural precursor cell proliferation", "regulation of Ras protein signal transduction", "positive regulation of dendrite extension", and "MAPK cascade" were also upregulated (Fig. 2d). In addition, a hierarchically clustered heatmap of various NSC-, neurogenesis-, and myelin-related genes showed elevated expression of each gene on both days 2 and 7 (Fig. 2e, Supplementary Fig. 2b-d).

Furthermore, downregulated genes in the day-2 and 7 HGF groups were related to inflammation-related GO terms (Fig. 2f, g, Supplemental Fig. 2e, f). These included "regulation of inflammatory response", "NIK/NF-kappaB signaling", and "microglial cell activation" on day 2, and “fibroblast proliferation”, “positive regulation of macrophage”, “glial cell activation” on day 7. Moreover, heatmaps associated with various proinflammatory-associated genes showed decreased expression of each gene in the day-2 HGF group compared with the control group (Fig. 2h). These results suggest that HGF administration in acute SCI could enhance neurogenesis, suppress the inflammatory response, and inhibit the fibrosis and gliosis at the molecular level.

### HGF pretreatment protects against spinal cord atrophy, promotes remyelination and enhances the survival of transplanted NS/PCs

**Fig. 3:**
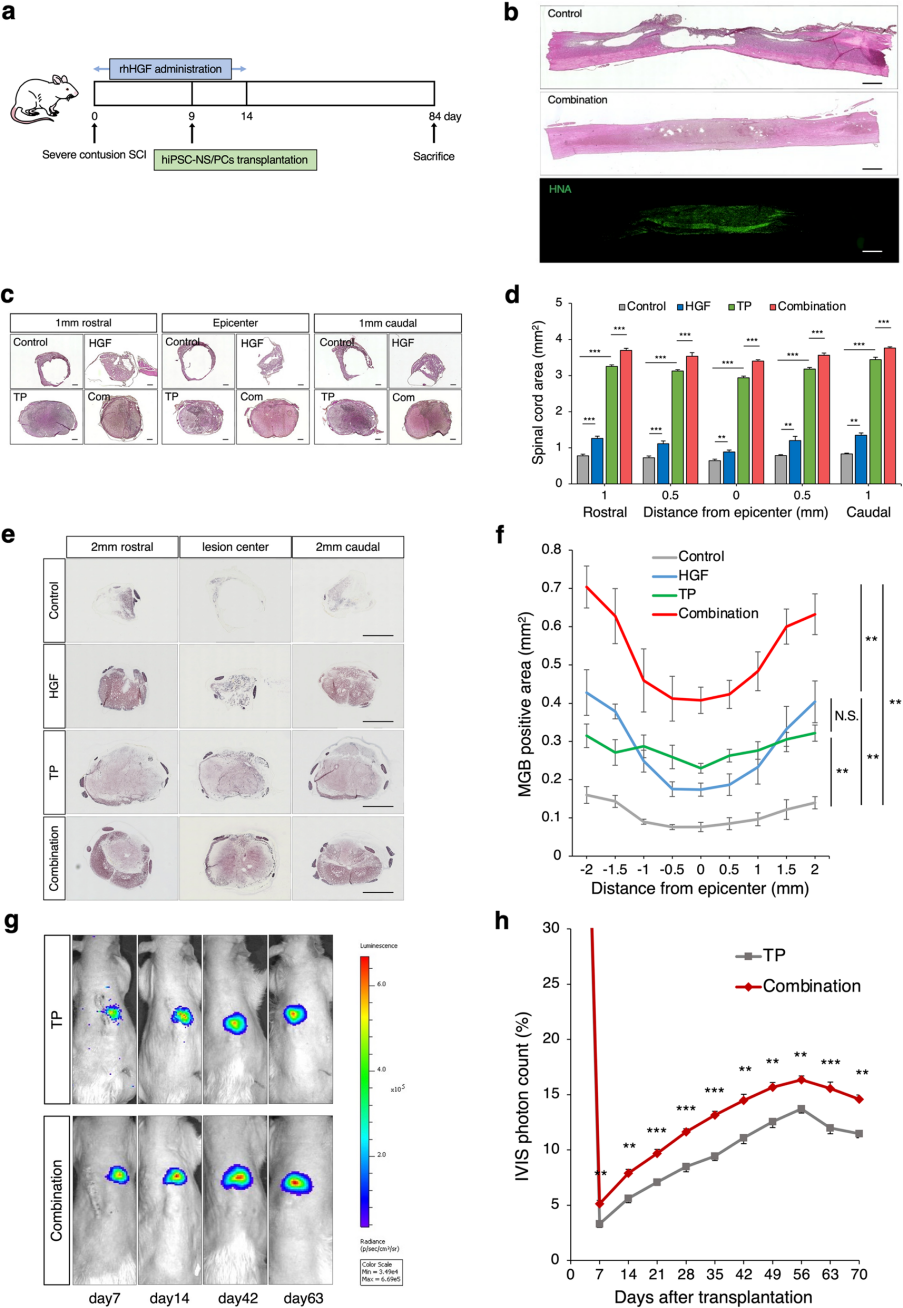
Spinal cord protection, remyelinating effects, and good survival of grafted NS/PCs through combination therapy. **a** Schematic of the time schedule for the combinatory therapy experiment. **b** Representative H&E-stained and immunohistochemical staining for HNA images of mid-sagittal sections in each group. Scale bars: 1000 μm. **c** Representative H&E-stained images of axial sections at the lesion epicenter and at the sites located 1 mm rostral and caudal in each group. Scale bar: 300 μm. TP: Transplantation group, Com: Combination group. **d** Quantitative analysis of the spinal cord area measured in H&E-stained axial sections at each level. *p < 0.05, **p < 0.01, ***p < 0.001, and N.S., not significant according to two-way measures ANOVA with the Tukey‒Kramer test. *n* = 6 in the control and HGF alone groups, *n* = 5 in the TP alone group, *n* = 6 in the Combination group. **e** Representative MGB-stained images of axial sections at the lesion epicenter and at the sites located 2 mm rostral and caudal in each group. Scale bar: 1000 μm. **f** Quantitative analysis of the myelinated area measured in MGB-stained axial sections at each level. *p < 0.05, **p < 0.01, ***p < 0.001, and N.S., not significant according to repeated-measures two-way ANOVA, followed by the Tukey‒Kramer test. *n* = 6 in the control and TP groups, *n* = 5 in the HGF group, *n* = 7 in the Combination group. Values are the mean ± SEM. **g** Representative images of the photon counts of BLI up to 63 days. **h** Quantitative analyses of the BLI-photon counts of the transplanted cells as percentage changes compared with those of the day of transplantation (TP group; *n* = 8, Combination group; *n* = 9). *p < 0.05, **p < 0.01, ***p < 0.001. Statistical analysis was performed using two-way ANOVA followed by the Tukey‒Kramer test. Values are the mean ± SEM

We found that HGF improved the microenvironment after acute SCI, so we next examined the effect of HGF in combination with hiPSC-NS/PC transplantation (Fig. 3a).

First, H&E staining of the spinal cord revealed that the control group exhibited significant cavitary lesions and atrophy, while the combination group showed a reduction in the injured lesion area (Fig. 3b). Additionally, human nuclear antigen (HNA) immunostaining demonstrated good engraftment of transplanted cells at the injury site (Fig. 3b). To examine atrophic changes in the injured spinal cord after each treatment, we performed quantitative analysis and identified significant differences in the cross-sectional area of the spinal cord among the control group, the TP group, and the HGF groups. These results suggest that HGF and hiPSC-NS transplantation suppressed atrophy of the injured spinal cord (Fig. 3c, d). In addition, we performed myelin gold black (MGB) staining to investigate myelination effect after each treatment. These also revealed significant differences between the combined group and the other three groups in terms of the MGB^+^ myelinated areas examined (Fig. 3e, f).

Next, we used a lentiviral method to transduce the hiPSC-NS/PCs with ffLuc, a fusion protein between the fluorescent protein Venus and firefly luciferase [25–27], to evaluate the efficacy of HGF pretreatment in promoting the survival of hiPSC-NS/PCs transplanted into the injured rat spinal cord using bioluminescence imaging (BLI) (Fig. 3g). The BLI analysis showed an increase in the photon counts of the transplanted hiPSC-NS/PCs from day 7 after cell transplantation, peaking at day 56. The survival rate at 56 days after transplantation in the combination group reached 16.3% ± 0.3%, which was higher than 13.7% ± 0.3% for the TP group (Fig. 3h; *p* < 0.01). These results indicate that HGF pretreatment enhances the viability of transplanted cells.

### Transplanted NS/PCs pretreated with HGF differentiate into three neuronal lineages and do not show tumorigenic changes

First, we assessed the neurospheres to be transplanted, which were fixed and identified as NS/PCs based on the presence of NSC markers in vitro (SOX2 and Nestin) (Supplementary Fig. 3a). Additionally, we analyzed the expression of c-Met and p–c-Met in neurospheres treated with rhHGF. Many cells expressing p–c-Met were observed in the HGF-treated neurospheres (Supplementary Fig. 3b). These results confirmed that rhHGF acts on hiPSC-NS/PCs.

**Fig. 4:**
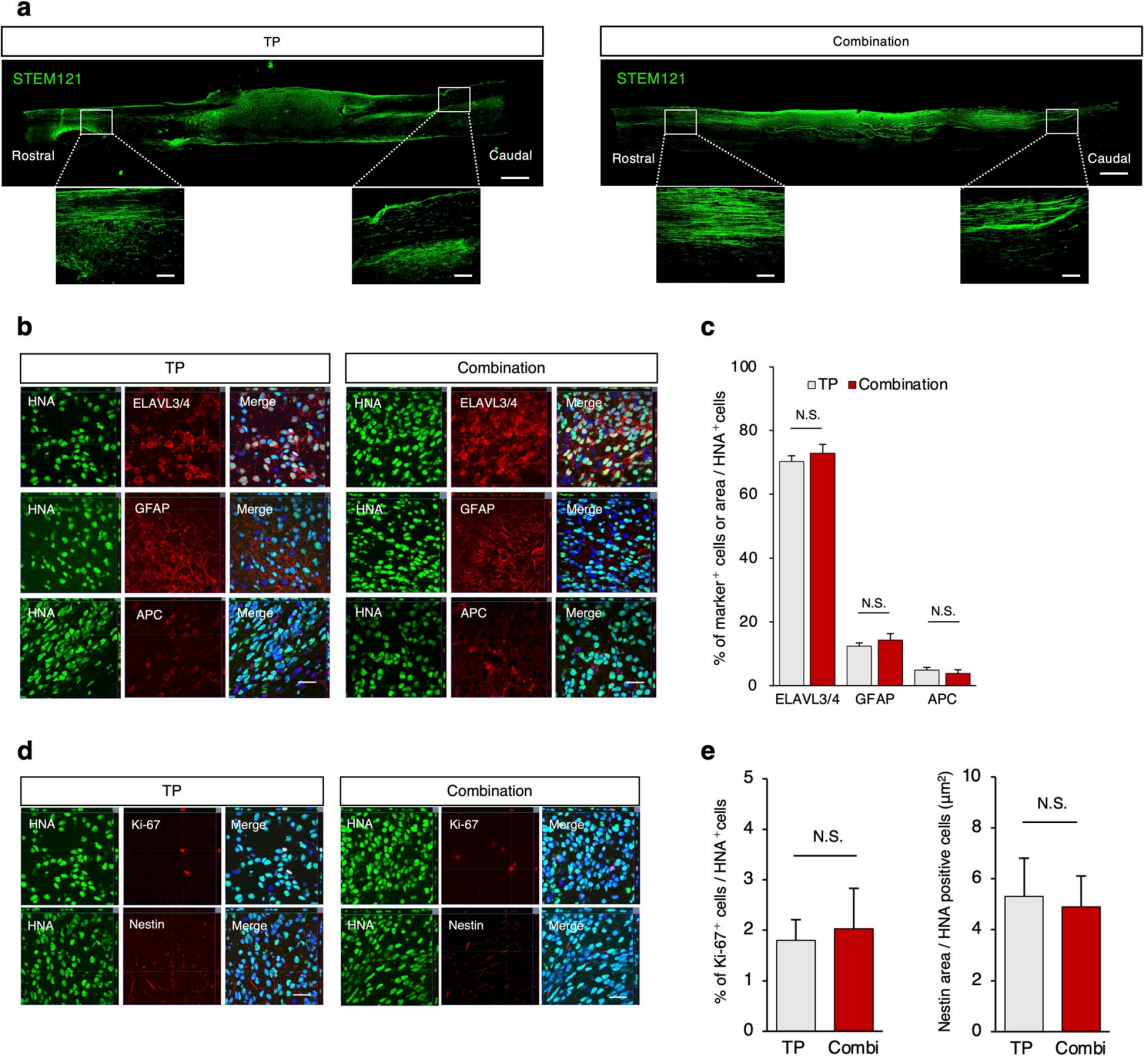
Migration and differentiation profile of the transplanted hiPSC/NS/PCs. **a** Representative image of a sagittal section stained for STEM121, which detects the human-specific cytoplasmic antigen in the TP and combination groups. STEM121 + grafted cells were elongated from the rostral and caudal sites. Scale bars, 1000 and 200 μm (enlarged images). **b** Representative images of neural cells differentiated from graft cells. HNA ^+^ grafted cells merged with ELAVL3/4 (neurons), GFAP (astrocytes), and APC (oligodendrocytes). Scale bar: 20 μm. **c** Quantitative analyses of the proportions of ELAVL3/4 ^+^, GFAP^+^, and APC^+^ cells among the HNA ^+^-grafted cells. *n* = 7 each. **d** Representative images of immunostaining for each group. HNA-positive transplanted cells were stained with Ki67 and Nestin. Nuclei were stained with Hoechst. Scale bar: 20 μm. **e** Quantitative analyses of proportions of Ki-67^+^ cells and Nestin^+^ area per HNA ^+^-grafted cells. *n* = 7 each. N.S., not significant according to the Mann‒Whitney U test. Values are the means ± SEMs for c and e

Second, immunohistological analyses were performed to evaluate the characteristics of the engrafted cells at 75 days after transplantation. STEM121, a specific human cytoplasm marker, demonstrated graft-derived nerve axons extending caudally and rostrally from the engrafted site in both the TP and combination groups (Fig. 4a). The neural differentiation of the transplanted cells was evaluated, and we found that the HNA-positive grafted cells differentiated into three neural lineages 75 days after transplantation (Fig. 4b): ELAVL3/4-positive neurons, GFAP-positive astrocytes, and APC-positive oligodendrocytes. There was no significant difference in the proportion of HNA^+^ cells immunopositive for cell-type-specific markers in either group (Fig. 4c).

Moreover, the number of immature cells, identified by the NSC marker Nestin or the cell proliferation marker Ki67, was low in both groups, indicating that HGF pretreatment did not induce tumorigenic changes in the graft-derived NS/PCs (Fig. 4d, e).

### The combined therapy promotes the regeneration of neuronal fibers and regrowth of raphespinal serotonergic fibers

**Fig. 5:**
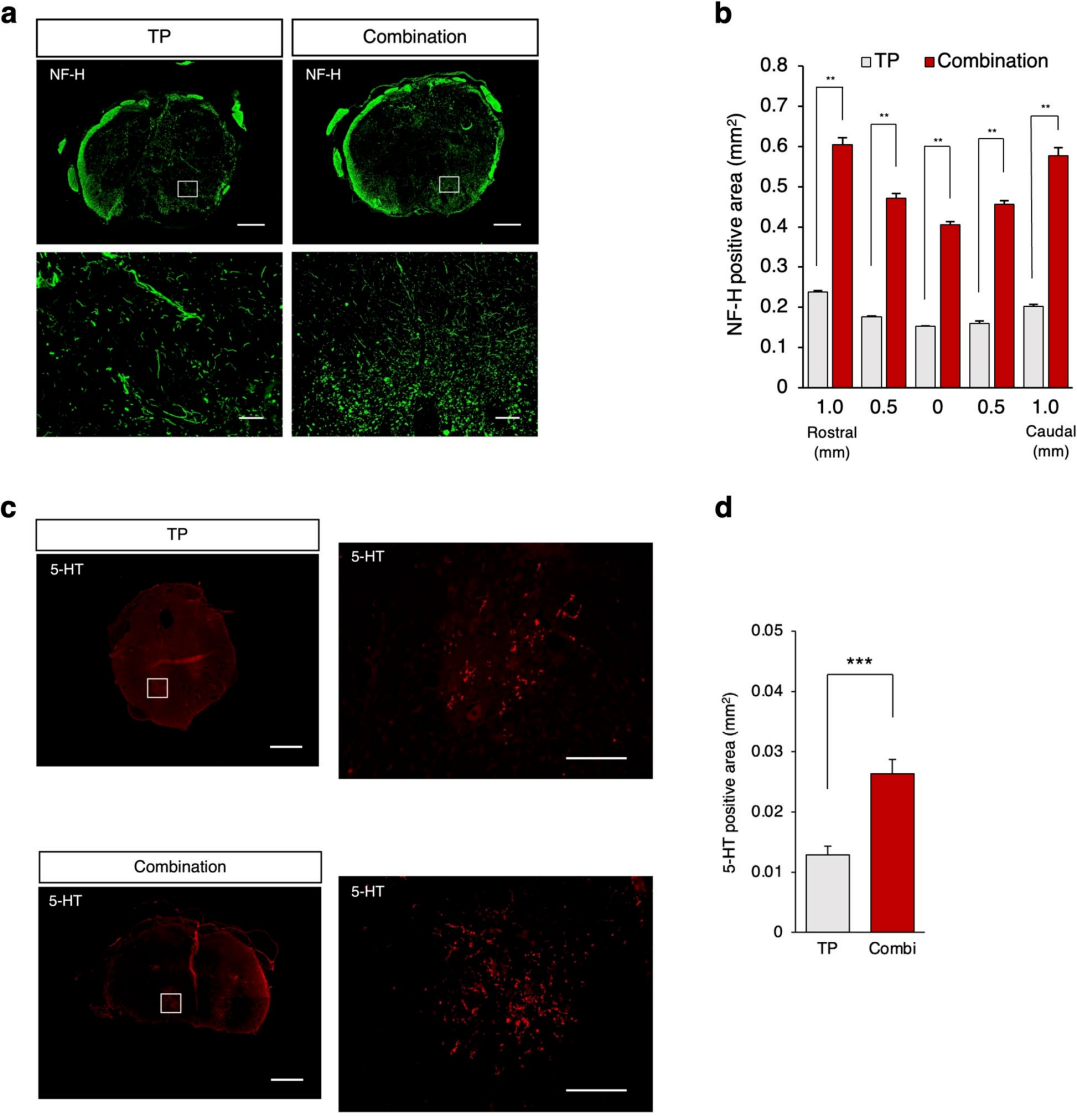
Combined therapy promoted regeneration of neuronal fibers and regrowth of raphespinal serotonergic fibers. **a** Representative image of axial sections stained for NF-H at the lesion epicenter and at the sites located 1 mm rostral and caudal in the TP and combination groups. Scale bars: 400 μm (upper) and 20 μm (lower). **b** Quantitative analysis of NF-H^+^ areas at each level. *n* = 5 each. **c** Representative images of immunostaining for 5-HT in axial Sects. 4 mm caudal to the epicenter. Scale bars: 100 μm (upper) and 50 μm (lower). **d** Quantitative analysis of the 5-HT-positive area (TP group; *n* = 7, control group; *n* = 7). Values are the mean ± SEM; *p < 0.05, **p < 0.01. Statistical analysis was performed using the Mann‒Whitney U test in (b, d). Combi: Combination group

To investigate the effects of combined therapy on neuronal regeneration after SCI, we performed immunohistochemical analyses using anti-NF-H antibodies (a marker of large-diameter neurofilaments). NF-H-positive neuronal fibers were more prominent in the combination group than in the TP group (Fig. 5a). We identified significant differences in the NF-H^+^ areas at all sites examined between the two groups (Fig. 5b).

Additionally, the regrowth of raphe spinal serotonergic fibers, which are believed to contribute to the recovery of motor function after SCI in rodents, was assessed using an anti-5-hydroxytryptamine (5-HT) antibody [28] (Fig. 5c). Quantitative analysis revealed that more 5-HT^+^ serotonergic fibers were observed in the combination group than in the TP group at the lumbar enlargement (Fig. 5d).

### Synapse formation between hiPSC-derived neurons and host neurons is spared by HGF pretreatment

**Fig. 6:**
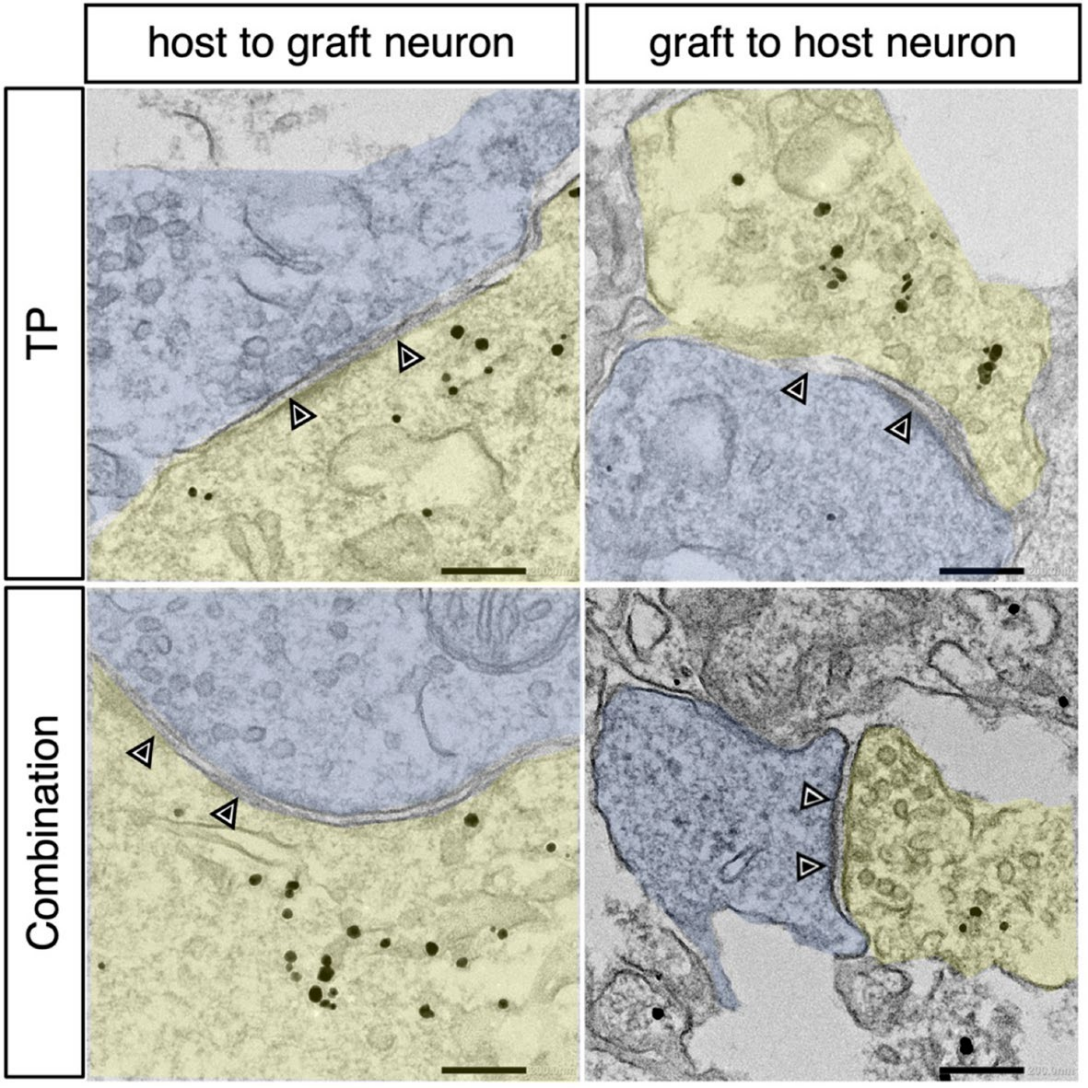
Synaptic formation between host and grafted neurons. Immunoelectron microscopy image of a synaptic connection (arrowheads) from immunogold-labeled STEM121-positive graft neurons (yellow) to host neurons (light blue) in the TP and combination groups. Scale bars: 200 nm

To assess the ability of transplanted cell-derived neurons to integrate with the host neural circuitry, we performed immunoelectron microscopy labeling with an anti-human cell cytoplasm antibody (STEM121). We observed pre- and postsynaptic structures and synapses between the STEM121^+^ transplanted cell-derived neurons and the host neurons in both groups (Fig. 6). These results suggest synapse formation between the transplanted cell-derived and spared host neurons.

### Combined therapy with HGF and hiPSC-NS/PC transplantation enhances motor functional recovery after SCI

**Fig. 7:**
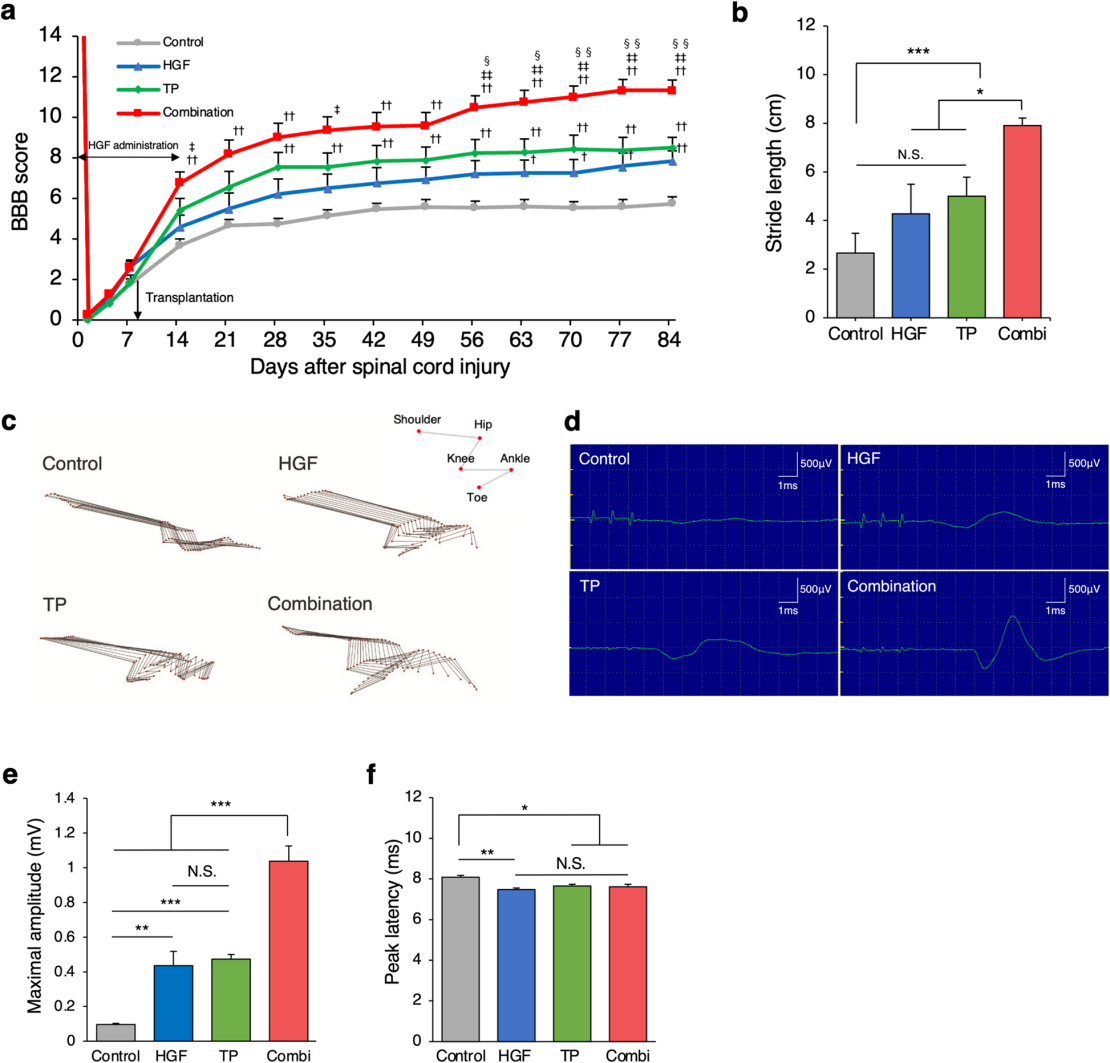
Acceleration of motor function recovery through combined therapy. **a** Serial BBB score assessment of hind limb motor functions among rats in the combination, TP, HGF, and control groups. The BBB scores were found to significantly improve in the combination group compared with the other groups. *n* = 16 for the combination group, *n* = 15 for the control and TP groups, *n* = 14 for the HGF group. **b** Analysis of stride length using the DigiGait system at 84 days after SCI (*n* = 12 for the combination group, *n* = 11 for the control and TP groups, *n* = 9 for the HGF group. **c** Kinematics analysis of representative stick diagrams from the four groups. **d** Representative images of MEP waves stimulated at the Th2 spinal cord and recorded from the distal quadriceps muscle tendon in the four groups. **e** Quantitative analysis of MEP maximal amplitude in the four groups. The latency was shorter in the treated three groups than in the control group. *n* = 4 each. **f** Quantitative analysis of MEP peak latency in the four groups. The maximal amplitude was significantly larger in the combination group than in the other groups. *n* = 4 each. The *p* values shown in (a) were calculated by repeated-measures two-way ANOVA, followed by the Tukey‒Kramer test. †, *p* < .05; ††, *p* < .01 versus the control; ‡, *p* < .05; ‡‡, *p* < .01 versus HGF alone; §, *p* < .05; §§, *p* < .01 versus TP alone. The *p* values shown in (b, e, f) were calculated by repeated-measures two-way ANOVA, followed by the Tukey‒Kramer test. *p < 0.05, **p < 0.01. N.S., not significant. Values are the means ± SEMs for (a, b, e, f). Combi: Combination group

We evaluated locomotor functional recovery using the Basso, Beattie, Bresnahan (BBB) Locomotor Rating Scale, treadmill gait analysis (DigiGait system), and kinematics analysis. The BBB scores indicated that the combination group exhibited significantly better functional recovery than the other groups at 56–84 days after SCI (Fig. 7a). Treadmill gait analysis revealed that the combination group (7.9 cm ± 0.3 cm) demonstrated a significantly better recovery in terms of stride length recovery than the other groups (Fig. 7b; combination vs. TP 5.0 cm ± 0.7 cm, *p* = 0.048: combination vs. HGF 4.2 cm ± 1.2 cm, *p* = 0.014: combination vs. control 2.6 cm ± 0.8 cm, *p* < 0.001). Qualitative data from the kinematic analysis revealed that the combined group demonstrated weight-supported gait and coordination (Fig. 7c). In addition, we carried out an electrophysiological analysis (motor evoked potential; MEP) to validate the motor circuit improvement (Fig. 7d). The amplitude showed a significant increase in the combination group (Fig. 7e; combination 1.03 mV ± 0.08 mV vs. TP 0.47 mV ± 0.02 mV, *p* < 0.001: combination vs. HGF 0.43 mV ± 0.08 mV, *p* < 0.001: combination vs. control 0.09 mV ± 0.01 mV, *p* < 0.001). Although the peak latency in the treated 3 groups (combination, TP, HGF) showed significant improvement compared to that of the control group, no significant differences were found among the three treated groups (Fig. 7f; combination: 7.61 ms ± 0.12 ms vs. TP: 7.65 ms ± 0.47 ms, *p* = 0.76: combination vs. HGF: 7.47 ms ± 0.07 ms, *p* = 0.99: TP vs. HGF, *p* = 0.60: combination vs. control: 8.08 ms ± 0.08 ms, *p* = 0.023: TP vs. control, *p* = 0.037: HGF vs. control, *p* = 0.004). Collectively, these data collectively suggest that combined therapy with HGF and hiPSC-NS/PCs enhanced locomotor functional recovery and motor circuit improvement.

## Discussion

In this study, we evaluated the efficacy of a combined treatment approach comprising HGF administration and transplantation of hiPSC-NS/PCs after SCI. Administering HGF following acute SCI not only had anti-inflammatory effects but also promoted tissue regeneration, including angiogenesis, neuroregeneration, and myelination. These regenerative effects played a critical role in preserving the damaged tissue, subsequently optimizing the microenvironment for transplantation and ultimately leading to more beneficial outcomes. Moreover, these environmental modulations improved the survival rates of transplanted cells from an early stage, indicating the synergistic histological benefits of combination therapy. These benefits include spinal cord protection and myelination, nerve fiber regeneration, and synaptic transmission, all of which contribute to improved locomotor function. Therefore, the combined approach of HGF administration and hiPSC-NS/PC transplantation presents a promising therapeutic strategy for SCI.

Our findings unveil the anti-inflammatory effects and immunomodulatory mechanisms induced by HGF administration following acute SCI. Comprehensive RNA sequencing analyses revealed that HGF inhibits the NF-κb signaling pathway, which influences a variety of immune cells, including neutrophils, macrophages, and microglia. Regarding microglial activation, histological examinations showed excess ballooning microglia at the injury site in the SCI group. These conditions can further amplify the inflammatory response due to the release of intracellular contents during cellular necrosis [29–31]. In contrast, the HGF-treated group retained a normal amoeboid phenotype and showed a decrease in the proliferation rate of activated microglia. These findings suggest that HGF treatment after SCI could mitigate excessive inflammatory responses by modulating microglial activity and cellular death. The suppression of acute inflammatory and immune responses likely plays an important role in modulating the spinal cord environment and preserving neurons after SCI.

Previous reports have suggested that HGF contributes to the maintenance of self-renewal and proliferation of NSCs by activating the MAPK/ERK pathway [14, 32]. In particular, our group has previously reported that p38MAPK can increase neurogenesis [33]. In the present study, HGF histologically increased the proliferation of endogenous NSCs by approximately twofold compared to that of the untreated group. Additionally, HGF enhanced the expression of stem cells and neurogenesis-related genes in host tissues, as shown by RNA sequencing. Therefore, we speculated that the increased endogenous NSCs differentiated into neuronal cells. This speculation is corroborated by previous studies demonstrating that HGF promoted the differentiation of NSCs into neurons and the formation of synapses between neurons and descending corticospinal fibers [34–36]. Thus, the results of this study suggest that HGF, with neuronal guidance signaling, could contribute to intrinsic neurogenesis after SCI.

The interactions between the vasculature and NS/PCs play a crucial role in neural regeneration [37–39]. Previous reports have suggested that vascular endothelial cell-derived humoral factors contribute to the proliferation and maintenance of NSCs [40]. In the pathological environment, vascular endothelial cells and NSCs collaboratively modulate neurogenesis [41–43]. Therefore, promoting vascular remodeling after SCI could enhance the survival of transplanted NS/PCs and nerve regeneration. In our study, HGF induced the proliferation of vascular endothelial cells, which led to an improvement in the survival of transplanted cells from the early stage. Furthermore, we found that surviving transplant-derived neurons promote nerve fiber regeneration in the lesion and connect synapses with host neurons. These results again emphasize the crucial role of neovascularization after SCI in neuroregeneration by transplanted NS/PCs.

The safety of iPSC-based SCI therapies, particularly their tumorigenicity, must always be considered [44]. HGF, as a promoter of cell proliferation [45], could potentially drive tumor-like hyperproliferation of the transplanted hiPSC-NS/PCs. However, before transplantation, we treated the hiPSC-NS/PCs with a γ-secretase inhibitor (GSI), which inhibits cell proliferation by blocking Notch signaling activation [46, 47]. The NS/PCs were exposed to HGF only for 5 days after transplantation in our experimental protocol. These procedures might prevent the tumorigenic changes of hiPSC-NS/PC transplantation therapy.

Our study demonstrated that the combination group showed improvements in locomotor parameters, resulting in an accelerated recovery of locomotor function compared to that of the group that received transplantation alone. Notably, a significant and precipitous recovery in the BBB score was observed in the combination group relative to the TP group at 49–56 days post-SCI (Fig. 7a). This trend was maintained until day 77 post-transplantation. These observations suggest that the surviving transplanted neurons gradually matured and subsequently established more efficient synaptic connections with the host neurons. These host neurons were maintained by HGF pretreatment, which was a critical step in the recovery of motor function. These data confirmed our previous report, as the number of mature neurons was found to gradually increase from 42 days after hiPSC-NS/PC transplantation, which is the time required for their maturation into neurons capable of eliciting spontaneous neuronal activity [1, 48]. The results of our study highlight the importance of preserving lesion tissue, including host neurons, to enhance the effect of cell transplantation therapy.

In conclusion, the combination therapy of HGF and hiPSC-NS/PC transplantation opens innovative possibilities as a two-phase treatment for acute to subacute SCI. This study suggests that pretreatment with HGF promotes the neuroregenerative process of NS/PC transplantation. For establishing optimal standardized treatment, a treatment protocol based on the severity of SCI should be established while consulting with the relevant government departments in each country. Therefore, further research is required to develop practical prognostic factors for SCI, including image diagnostics and biomarkers. The efficacy of each of these treatments will be further confirmed in future large-scale human clinical trials, potentially establishing a new standard of care for the treatment of SCI.

## Methods

### Animals

Adult (eight-week-old) female athymic nude rats (F344/NJcl-*rnu*/*rnu*, weight = 110–180 g, CLEA Japan, Inc., Tokyo, Japan) were used in these experiments. The rats were housed randomly in groups of three or four per cage (24 × 42 × 24 cm) regardless of the experimental group. The animals were kept on a 12/12 h light/dark cycle in an environment with controlled temperature and humidity and provided ad libitum access to food and water. Antibiotics (orbifloxacin; Sumitomo Dainippon Pharma Animal Health, Inc., Osaka, Japan) were injected for 3 days after SCI and other surgeries. All experimental procedures were approved by the Experimental Animal Care Committee of KEIO University, School of Medicine (assurance no. 13020) and performed per the Guide for the Care and Use of Laboratory Animals (National Institutes of Health, Bethesda, MD). In this study, all rats were anesthetized by subcutaneous injection of 0.4 mg/kg medetomidine hydrochloride, 2 mg/kg midazolam, and 2.5 mg/kg butorphanol.

### NS/PC-derived human iPSC culture

The YZWJs513 hiPSC line was used throughout this study. NS/PCs were prepared at a Good Manufacturing Practice (GMP)-grade cell processing facility at Osaka National Hospital, Japan. The clinical-grade human leukocyte antigen superdonor-derived, integration-free hiPSC line YZWJs513, established by the iPSC Stock Project and organized by the Kyoto University (CiRA) for iPS Cell Research and Application, was used. For in vivo analyses, the cells were cultured for 5 days in a floating culture, and the neurospheres were used for transplantation. Cells were treated with the small molecule GSI and N-[N-(3,5-difluorophenacetyl)-l-ananyl]-S-phenylglycine t-butyl ester (DAPT) (10 μM, Sigma-Aldrich, Inc., D5942) for 1 day before transplantation, as described in a previous study [4].

### Preparation of osmotic mini-pump containing rhHGF

Osmotic mini pumps (Alzet model 2002; nominal pumping rate 0.5 μl/hr, nominal duration 2 weeks, nominal reservoir 200 μl, Alzet, CA, USA) were filled with PBS or with rhHGF (Kringle Pharma, Inc., Osaka, Japan) diluted with dilution medium (10 mM citrate buffer, 0.3 M NaCl, 0.01% polysorbate 80, pH 6.0) so that each pump contained 200 μg of rhHGF. Each pump was connected to an intrathecal catheter (rat Intrathecal Catheter short; Alzet, CA, USA), and the apparatuses were incubated in sterile PBS at 37 °C for 12 h before use.

### Severe contusive SCI, intrathecal infusion of rhHGF and NS/PC transplantation

Severe contusive SCI was induced at the level of the tenth thoracic spinal vertebra using an Infinite Horizon impactor (240 kdyn; Precision Systems and Instrumentation, Fair-fax Station, VA, USA) with a 2 mm tip, as described previously [49]. This force was chosen because pilot studies revealed that milder (220 kdyn) injuries allowed the animals to walk via their hindlimbs over time, thus failing to recreate the recovery course of severe SCI. For SCI at the T10 level, a T12 laminectomy was carried out, and an intrathecal catheter was inserted from the T12 level to the T10 level. An osmotic mini pump filled with 200 μg rhHGF (HGF group) or PBS (control group) was connected to a catheter and placed in the subcutaneous space on the middle side of the animal’s back. The pump was left in place to deliver a 200 μg total dose of rhHGF for 2 weeks. The pump was removed 2 weeks after administration. The rats with SCI were randomly assigned to each group. For the sham group, the same surgical procedure was performed as for the SCI experimental groups; however, no SCI was inflicted.

For TP group rats (combination group: *n* = 16, 1 rat died; TP group: *n* = 14, 3 rats died), 9 days after injury, hiPSC-NS/PCs (1 × 10^6^ cells in 2 μl of phosphate-buffered saline [PBS]) were transplanted into the lesion epicenter of each rat with a Hamilton syringe with a 27G metal needle using a micro stereotaxic injection system (KDS310; Muromachi-Kikai Co., Ltd.). An equal volume of PBS was similarly injected into PBS group rats (HGF group: *n* = 15, 2 rats died; control group: *n* = 15, 2 rats died) in place of the hiPSC-NS/PCs. The injected depth was 0.4 to 1.0 mm, and the injection speed was 1 μl/min.

### Transcriptome analysis

Spinal cord samples collected 2 or 7 days after SCI were used for analysis, and samples were pooled in the control (PBS) and HGF (HGF-treated) groups (*n* = 4 each, total: *n* = 16). Then, 3 mm-long dissected spinal cords were rapidly frozen, and total RNA was isolated using the RNeasy Mini Kit (Qiagen) following the manufacturer’s instructions with slight modifications. Total RNA from each sample was purified, and mRNA libraries were prepared according to the TruSeq Stranded mRNA LT Sample Prep Kit (Illumina, San Diego, CA, United States) protocol and sequenced using NovaSeq 6000 (Illumina) to obtain paired-end reads. Raw reads were trimmed based on the read quality and read length using Trimmomatic 0.38. After read mapping to the reference genome (rn6), StringTie version 2.1.3b was used for transcript count. The read count of each sample was normalized to fragments per kilobase of transcript per million mapped reads (FPKM) and transcripts per kilobase million (TPM).

Differentially expressed gene (DEG) analysis was performed on 2 comparison pairs as requested using edgeR. The raw count data were normalized with the trimmed mean of M-values (TMM) method in the edgeR package. Statistical analysis was performed using edgeR with Fold Change, exactTest for each comparison pair. The significant results were selected under the conditions of |FC|≥ 2 and exact test raw *p* value < 0.05. An enrichment test based on the GO (http://geneontology.org/) DB was conducted with a significant gene list using the g:Profiler tool (https://biit.cs.ut.ee/gprofiler/). For GO analysis, adjusted *p* values were calculated by Benjamini‒Hochberg tests (FDR: false discovery rate).

R software version 4.2.3 was utilized to generate gene expression heatmaps. The normalized expression values of the selected genes were used as input data. Z scores were calculated for each gene across the samples to compare gene expression profiles. The resulting Z score matrix was visualized as a heatmap. Color gradients were assigned to indicate relative gene expression levels, with warmer colors representing higher expression and cooler colors representing lower expression.

### Behavioral analyses

Hindlimb locomotor function was evaluated weekly until 12 weeks after SCI using the BBB scale [50]. Two investigators blinded to the groups performed the behavioral analyses. The stride length of the hindlimbs on a treadmill was measured at a speed of 5 cm/s (Rat Specifics, Inc., Framingham, MA, USA). Briefly, the animal’s gait was video recorded with the treadmill system. The footsteps were manually marked in the video frames and then subjected to analysis to evaluate the gait of the rats. Kinematics analysis was performed using four cameras (Go Pro HERO5 Black CHDX-502) to record multiple movements of the shoulders and hindlimbs [2]. The bilateral shoulders, hips, knees, ankles, and toes were labeled, and the images were analyzed using KinemaTracer software (Kissei Comtec).

### Tissue processing and histological analyses

The spinal cord was dissected and postfixed in 4% PFA and was then embedded in frozen section compound. The tissue sections were stained with the following primary antibodies: c-Met (mouse IgG1, 1:300, Cell Signaling Technology, Inc., 3127), phosphorylated c-Met (rabbit IgG, 1:100, Invitrogen, Inc., 44-882G), RECA1 (mouse IgG1, 1:200, Santa Cruz Biotechnology, Inc., sc-52665), Iba1 (rabbit IgG, 1:250, Wako, Inc., 01919741), GFAP (rabbit IgG, 1:500, Proteintech, Inc., 16,825–1-AP; mouse IgG2a, Thermo Fisher, Inc., 13–0300), SOX2 (goat IgG, 1:50, R&D Systems, Inc., AF2018), HNA (mouse IgG1, 1:100, Millipore, Inc., AB1281), STEM121 (mouse IgG1, 1:200, TaKaRa Bio, Inc., Y40410), ELAVL3/4 (mouse IgG2b, 1: 100, Molecular Probes, Inc., A-21271), APC (mouse IgG2b, 1:300, Abcam, Inc., ab16794), Ki67 (rabbit IgG, 1:1000, Leica, Inc., NCL-Ki67p), Nestin (rabbit IgG, 1: 500, IBL, Inc., 18,741), NF–H (200 kDa) (mouse IgG1, 1:200, Sigma-Aldrich, Inc., MAB5262), 5-HT (goat IgG, 1:500, Immunostar, Inc., 20,079). Then, the sections were incubated with Alexa Fluor-conjugated secondary antibodies (1:1000) and Hoechst 33,258 (10 μg/ml, Sigma-Aldrich). H&E staining and MGB staining were performed with the Black Gold II Ready-to-Dilute (RTD) Staining Kit (Biosensis, SA, Australia). Images were acquired at room temperature using standard filter sets with a fluorescence microscope (BZ-X710; Keyence, Osaka, Japan) and a confocal laser-scanning microscope (LSM 780; Carl Zeiss, Jena, Germany), and the acquisition settings were kept constant for all groups for each experiment. Quantitative analysis of all images was performed using Zen 2012 SP5 software (version 14.0.0.0; Carl Zeiss) or ImageJ software (version 13.0.6/1.53 k).

### EdU labeling and staining

EdU labeling was performed with an EdU Click 647 Kit (Base Click, Munich, Germany) according to the manufacturer's protocol. Nude rats were intraperitoneally injected with EdU (50 mg/kg/day) daily after SCI. The injections started the day after SCI and continued until 7 days, with the last injection occurring 4 h before sacrifice. Immunohistochemical staining for EdU was conducted according to the manufacturer's protocol.

### In vivo imaging (bioluminescence imaging) of transplanted cells

For confirmation of the survival of the transplanted hiPSC-NS/PCs, a Xenogen-IVIS spectrum-cooled charge-coupled device (CCD) optical macroscopic imaging system (Caliper Life-Science, Hopkinton, MA, USA) was used for bioluminescence imaging (BLI) [51]. Monitoring was performed once per week following cell transplantation. Eight rats in the TP group and nine rats in the Combination group were examined for in vivo imaging. D-Luciferin (VivoGlo Luciferin; Promega, Madison, WI) was intraperitoneally injected at a dose of 800 mg/kg body weight. Animals were placed in a light-tight chamber, and photons emitted from luciferase-expressing cells were collected with integration times of 5 s to 2 min, depending on the intensity of bioluminescence emission. BLI signals were quantified in maximum radiance units [photons per second per centimeter squared per steradian (p/s/cm2/sr)] and are presented as log10 (photons per second) values.

### Immunoelectron microscopy analysis

Immunoelectron microscopy analysis was performed on spinal cord sections. The detailed immunoelectron microscopy procedure was performed as described in a previous study [52]. In brief, spinal cord tissues were perfused and postfixed with 4% PFA for 12 h, followed by cryoprotective treatment with 15% and 30% sucrose. Frozen tissue blocks in cryocompound were sectioned at 20 μm thicknesses using a cryostat (CM3050S, Leica Microsystems, Wetzlar, Germany). Sections were incubated with 5.0% Block Ace solution (DS Pharma Promo) containing 0.01% saponin in 0.1 M PB for 1 h, then incubated with primary mouse anti-human cytoplasm antibody (STEM121, 1:200, TaKaRa Bio, Inc., Y40410) for 72 h at 4 °C, followed by incubation with FluoroNanogold-conjugated goat anti-mouse secondary antibody (1:100, Thermo Fisher Scientific, Inc.) for 24 h at 4 °C. After 2.5% glutaraldehyde fixation in 0.1 M PB, nanogold signals were enhanced with silver enhancement solution for 2.5 min at 25 °C. Gold-labeled sections were postfixed with 1.0% OsO_4_ for 90 min at 4 °C, *en bloc* stained with uranyl acetate for 20 min at 25 °C, dehydrated through a graded ethanol series and embedded into pure Epon. Ultrathin Sects. (80 nm) were prepared with an ultramicrotome (UC7, Leica Microsystems) and stained with uranyl acetate and lead citrate. The sections were imaged by transmission electron microscopy (JEM1400 plus, JEOL, Tokyo, Japan).

### Electrophysiology

Electrophysiological experiments (MEP: motor-evoked potential) were performed using a Neuropack S1 MEB9402 signal processor (Nihon Kohden, Tokyo, Japan) 84 days after SCI (*n* = 4 each). The surface of the T2 spinal cord was stimulated, and needle electrodes were used to record the signal from the hindlimb. An active electrode was placed in the quadriceps muscle, a reference electrode was placed near the distal quadriceps muscle tendon, and a ground electrode was placed in the back muscle. The stimulus parameters were an intensity of 2.0 mA, a duration of 0.2 ms, and an interstimulus interval of 1 Hz. The maximal amplitude was measured in a peak-to-peak manner. Peak latency was measured as the length of time from the stimulation to the highest point of the MEP wave.

### Neural differentiation analysis in vitro

The hiPSC-NS/PCs cells were cultured for 5 days in a floating culture, and the neurospheres were administered rhHGF (20 ng/per) or PBS. After fifteen minutes, it embedded in iPGell (PG20-1; Genostaff, Tokyo, Japan) following the manufacturer’s instructions, and frozen and sectioned at a 16 μm thickness on a cryostat (CM3050S; Leica Microsystems, Wetzlar, Germany). Tissue sections were stained with the following primary antibodies: anti-SOX2 (mouse IgG2a, 1: 40, RD systems, Inc., MAB2018), anti-Nestin (mouse IgG1, 1: 200, Millipore, Inc., MA5326), anti-c-Met (mouseIgG1, 1: 200, Cell signaling, Inc., 25H2), anti-phosphorylated c-Met (rabbit IgG, 1:100, Invitrogen, Inc., 44-882G). All samples were incubated overnight at 4 ℃ and then incubated with Alexa Fluor-conjugated secondary antibodies (1: 500, Thermo Fisher Scientific, Inc.) for 1 h at room temperature. Nuclei were stained with Hoechst 33,258 (10 μg/ml, Sigma-Aldrich), then examined under a confocal laser-scanning microscope (LSM 700, Carl Zeiss, Jena, Germany).

### Quantification and statistical analyses

Statistical analysis was performed with SPSS Statistics (version 28.0.1.0; Japan IBM, Tokyo, Japan). All data are presented as the mean ± SEM. The Mann‒Whitney U test was used for comparison between the 2 groups. One-way ANOVA followed by the Tukey–Kramer test was applied for group analysis. Two-way ANOVA followed by the Tukey–Kramer test was used for group and behavioral analyses. Differences were considered significant at *p < 0.05, **p < 0.01, ***p < 0.001.

### Supplementary Information

**Additional file 1.** 

**Additional file 2.** 

**Additional file 3.**

## Data Availability

RNA-seq data have been deposited in the Gene Expression Omnibus (GEO) under accession. All data are available from the corresponding author on reasonable request.
