# Supplementary figures and images for: Hepatocyte growth factor pretreatment boosts functional recovery after spinal cord injury through human iPSC-derived neural stem/progenitor cell transplantation
Source: Inflamm Regen. 2023 Oct 16;43:50. doi: 10.1186/s41232-023-00298-y (PMC10577910; doi:10.1186/s41232-023-00298-y)

Supplemental Fig.1

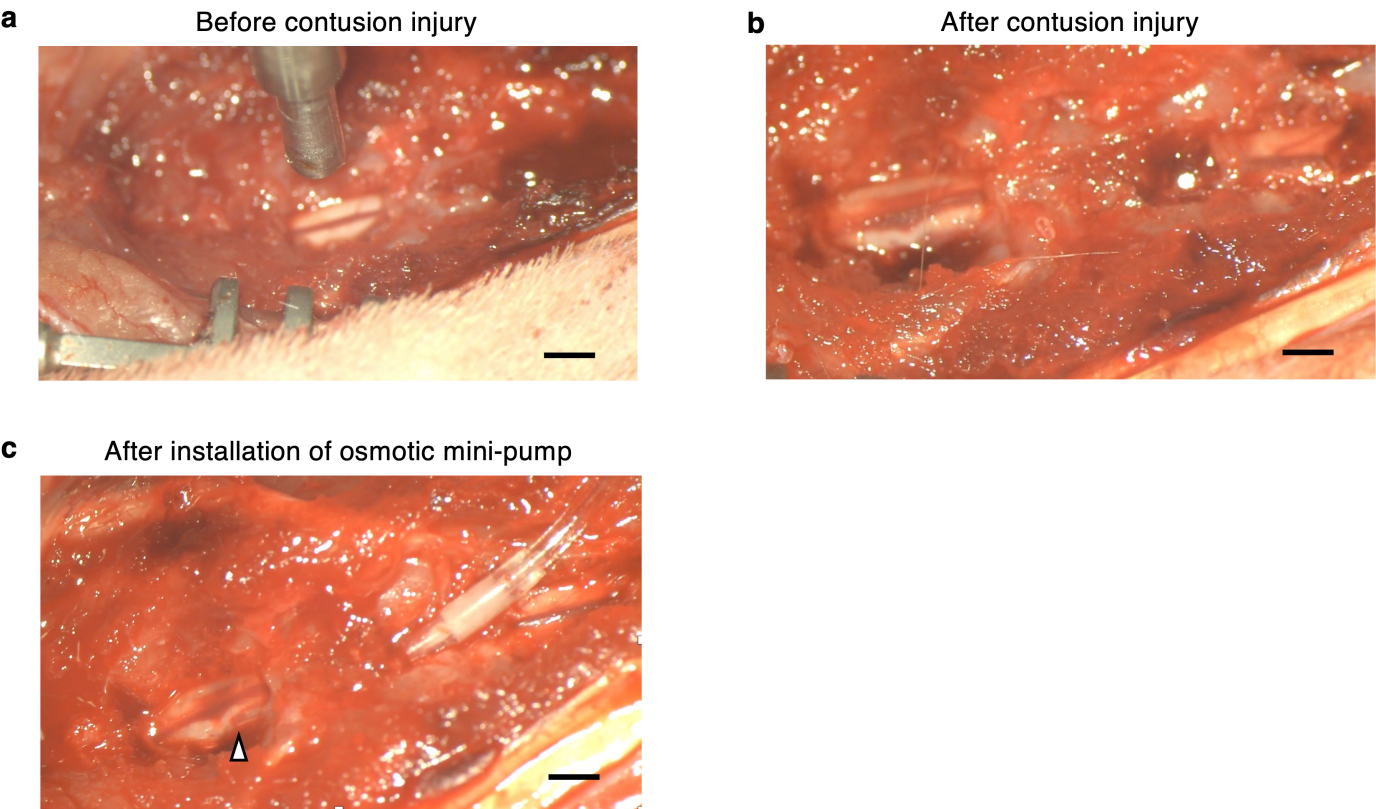

Supplement: Supplementary file 1 — Additional file 1. [file 41232_2023_298_MOESM1_ESM.pdf]

Supplemental Fig.2

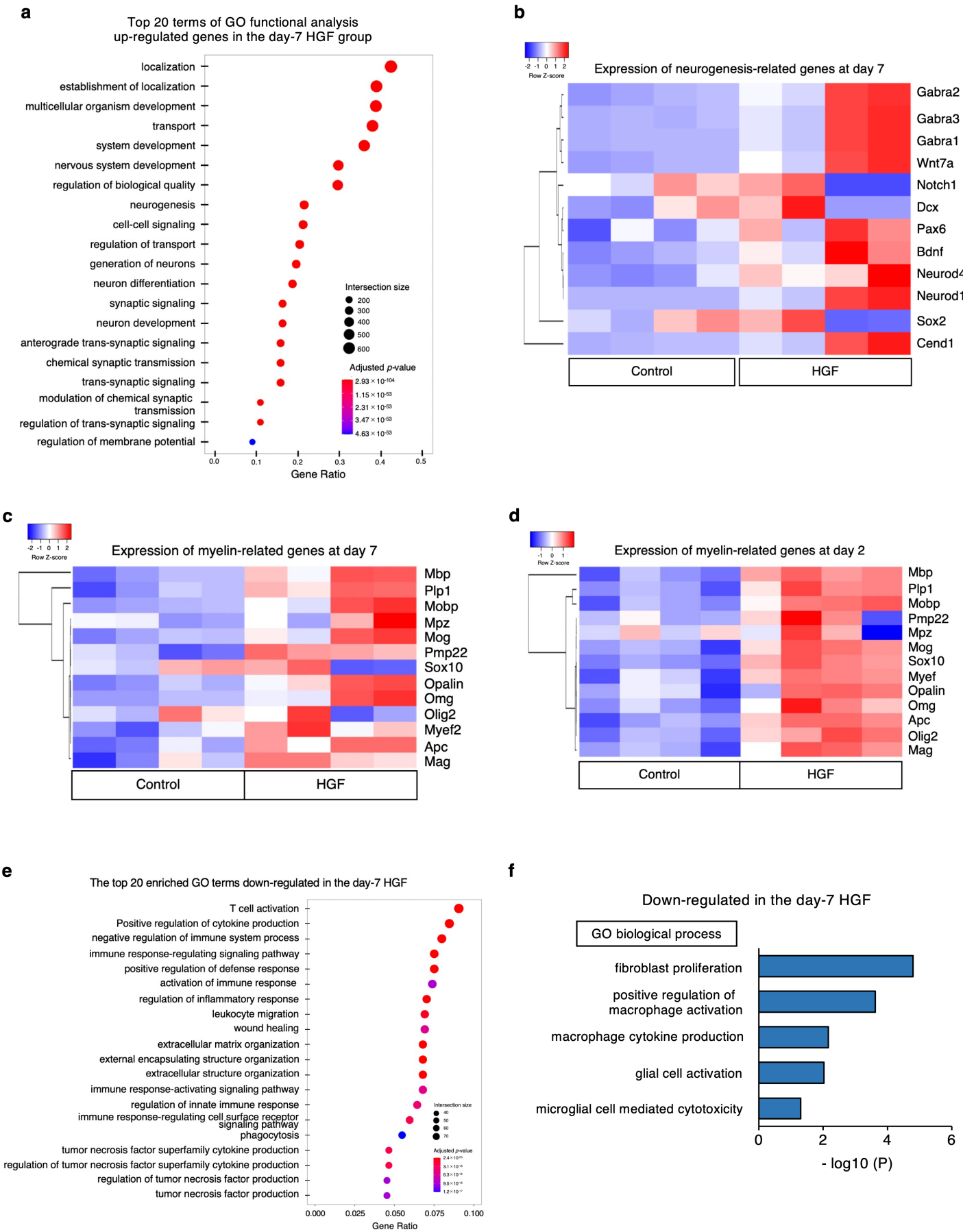

Supplement: Supplementary file 2 — Additional file 2. [file 41232_2023_298_MOESM2_ESM.pdf]

Supplemental Fig.3

a

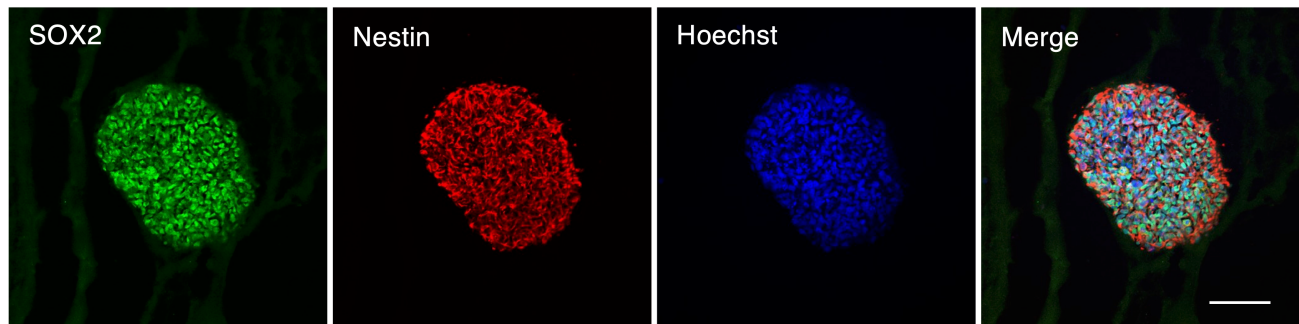

b

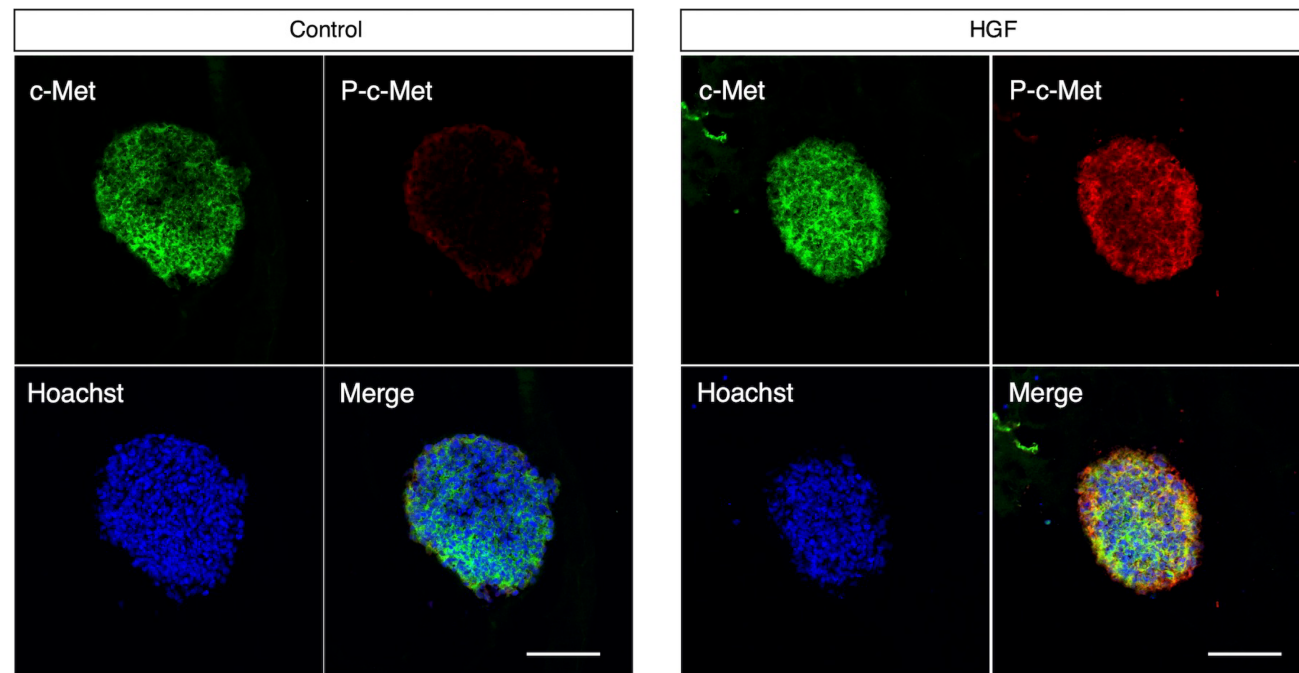

Supplement: Supplementary file 3 — Additional file 3. [file 41232_2023_298_MOESM3_ESM.pdf]
